# UCP1-Dependent Thermogenic Adipose Tissue in Human Disease: Adipose-Centered Mechanisms, Biomarker Limitations, and Translational Perspectives

**DOI:** 10.3390/ijms27146416

**Published:** 2026-07-19

**Authors:** Man Yan, Hengping Wang, Wanli Sha, Ying Fu

**Affiliations:** 1 Technology Innovation Center of Pig Ecological Breeding and Disease Prevention and Control, Jilin Agricultural Science and Technology College, Jilin City 132101, China; jlyanjingyu@163.com; 2 Jilin Collaborative Innovation Center for Antibody Engineering, Jilin Medical University, Jilin City 132013, China; wanghengping0116@163.com

**Keywords:** UCP1, brown adipose tissue, beige adipocytes, thermogenesis, mitochondrial uncoupling, biomarkers, obesity, diabetes, inflammation, cancer metabolism

## Abstract

Uncoupling protein 1 (UCP1) is a mitochondrial inner-membrane carrier classically recognized as the molecular effector of non-shivering thermogenesis in brown adipose tissue. By dissipating the proton-motive force generated by oxidative phosphorylation, UCP1 converts stored chemical energy into heat and enables adaptive thermogenesis during cold exposure. The rediscovery of metabolically active brown adipose tissue (BAT) and inducible beige adipocytes in adult humans has renewed interest in UCP1-positive thermogenic adipose tissue as a regulator of systemic metabolism and a potential target for therapeutic modulation in obesity and cardiometabolic disease. Beyond thermogenesis, accumulating evidence indicates that UCP1-positive brown/beige adipocytes and thermogenic adipose tissue are involved in lipid and glucose metabolism, mitochondrial redox homeostasis, inflammatory remodeling, organ protection, and tumor-associated metabolic adaptation, mainly through adipocyte-autonomous mechanisms and adipose–organ communication. However, UCP1 biology is complex: BAT activity measured by imaging does not directly quantify UCP1 proton conductance, non-adipose UCP1 expression is often low and technically challenging to validate, local cell-autonomous UCP1 function in non-adipose tissues remains controversial, and UCP1-independent thermogenic pathways may compensate in selected contexts. In this review, we summarize the molecular and structural basis of UCP1 function, its regulation at transcriptional and post-transcriptional levels, and its biological roles in cellular and systemic homeostasis, with explicit distinction between direct adipocyte-autonomous UCP1 functions, indirect systemic effects mediated by thermogenic adipose tissue, and preliminary or incompletely validated evidence of local UCP1 activity in non-adipose cells. We further discuss the association of UCP1-positive thermogenic adipose tissue with obesity, type 2 diabetes mellitus (T2DM), cardiovascular disease, kidney injury, liver disease, neurological disorders, and cancer. Finally, we evaluate UCP1-related thermogenic adipose tissue activity as a biomarker and therapeutic target, highlighting current limitations, safety concerns, and future directions for precision metabolic medicine.

## 1. Introduction

Uncoupling protein 1 (UCP1) is a mitochondrial inner-membrane carrier best known for its essential role in non-shivering thermogenesis in brown adipose tissue (BAT). By dissipating the proton-motive force generated by the respiratory chain, UCP1 uncouples substrate oxidation from ATP synthesis and converts chemical energy into heat. This canonical function enables mammals to maintain body temperature during cold exposure and has long positioned UCP1 as a central effector of adaptive thermogenesis [1,2,3].

The renewed interest in UCP1 stems from the recognition that metabolically active brown and beige adipocytes persist in adult humans and are inversely associated with obesity, insulin resistance, and cardiometabolic risk [4,5,6,7,8]. Unlike rodents, in which classical interscapular BAT is prominent, adult humans mainly harbor brown/beige-like adipocytes in supraclavicular, cervical, paravertebral, mediastinal, and perirenal depots. This anatomical and developmental heterogeneity makes direct extrapolation from mouse BAT studies to humans challenging.

In parallel, advances in metabolic imaging, mitochondrial bioenergetics, single-cell transcriptomics, and structural biology have substantially expanded our understanding of UCP1 regulation and function. UCP1 is now recognized not only as a thermogenic protein but also as a regulator of lipid and glucose metabolism, mitochondrial redox balance, inflammatory signaling, and interorgan metabolic communication [9,10,11,12]. Importantly, UCP1-dependent thermogenesis coexists with UCP1-independent mechanisms, including creatine-driven substrate cycling, calcium cycling, futile lipid cycling, and other mitochondrial pathways [13,14,15].

Emerging evidence further links altered UCP1 expression or activity to a broad spectrum of human diseases, including obesity, type 2 diabetes mellitus (T2DM), cardiovascular disease, renal injury, liver steatosis and fibrosis, neurodegeneration, and cancer. These findings raise the possibility that UCP1 may serve as both a biomarker of metabolic health and a therapeutic target. However, translating UCP1 biology into clinical interventions remains challenging because of species differences, tissue heterogeneity, compensatory thermogenic pathways, and safety concerns associated with systemic mitochondrial uncoupling.

In this review, we summarize recent advances in the molecular structure, tissue distribution, regulatory mechanisms, and biological functions of UCP1. We then discuss its emerging roles in metabolic and organ-specific diseases, evaluate its potential as a biomarker, and highlight therapeutic strategies and unresolved questions relevant to precision metabolic medicine.

## 2. Molecular and Structural Basis of UCP1 Function

### 2.1. Gene, Protein Architecture, and Mitochondrial Carrier Features

**Figure 1:**
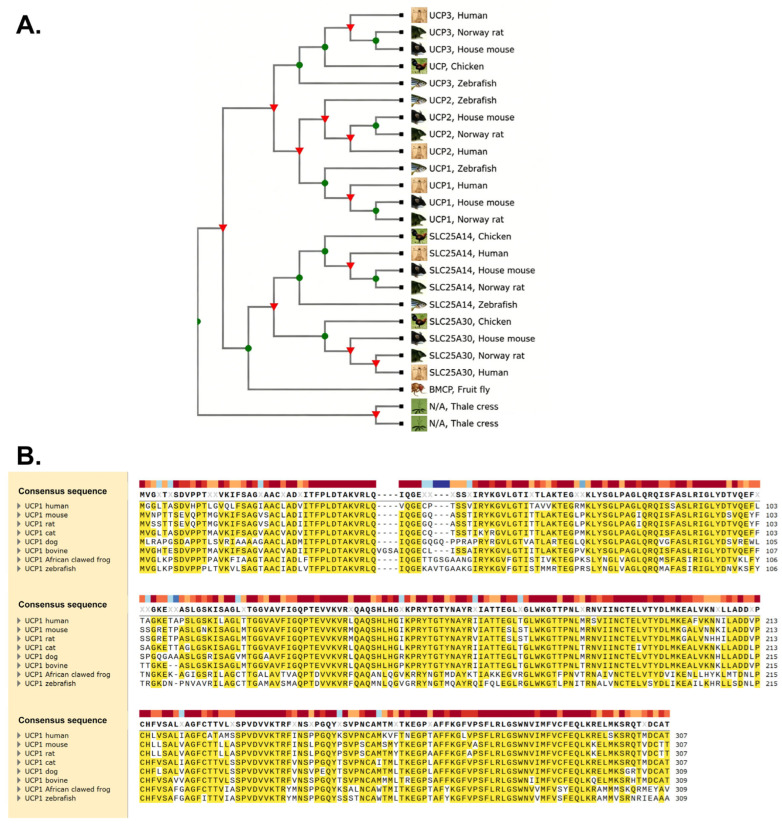
Phylogenetic relationships of uncoupling protein (UCP) family homologues and cross-species conservation of UCP1. (**A**) Phylogenetic tree of the UCP family and related SLC25 homologues. This figure was adapted and visualized based on the curated gene family tree from the TreeFam database (Family ID: TF323211; https://www.treefam.org/family/TF323211, accessed on 12 May 2026), which contains 314 homologous sequences from 94 species. The original sequence alignment was performed using MCoffee, and the gene tree was constructed by TreeBest integrating neighbor-joining (NJ) and PhyML algorithms, followed by reconciliation with the species tree to optimize topology. The authors selected representative taxa from key evolutionary branches for display and layout optimization. An *Arabidopsis thaliana* sequence is used as the outgroup for tree rooting. Protein names and species of origin are indicated at the terminal branches, and species icons are used to distinguish vertebrate groups. Node symbols (red triangles and green circles) denote key evolutionary branches and illustrate the phylogenetic relatedness among different homologues. (**B**) Multiple sequence alignment of UCP1 proteins across species. The aligned sequences include UCP1 homologues from *Homo sapiens* (P25874), *Mus musculus*(P12242), *Rattus norvegicus* (P04633), *Felis catus* (A0ABI7VRL1), *Canis lupus familiaris* (Q9GMZ1), *Bos taurus* (P10861), *Xenopus laevis* (A0A6I8PZS2), and *Danio rerio* (Q7ZVP4). Sequences were obtained from the UniProt database (https://www.uniprot.org/, accessed on 19 May 2026), and multiple sequence alignment and visualization were performed using SnapGene software (Version 8.0). The consensus sequence is shown above the alignment. Background shading and the upper colored bars indicate amino acid conservation and physicochemical similarity, respectively. Numbers on the right denote amino acid positions, highlighting evolutionarily conserved regions of UCP1.

Human UCP1 is located on chromosome 4q31 and encodes a mitochondrial inner-membrane protein of approximately 33 kDa. UCP1 belongs to the solute carrier family 25 (SLC25) mitochondrial carrier family and shares the canonical tripartite architecture of mitochondrial carriers, containing six transmembrane α-helices organized as three homologous repeats [16,17]. Like other mitochondrial carriers, UCP1 contains conserved sequence motifs that contribute to substrate or ligand interaction, conformational transitions, and membrane embedding. Phylogenomic analysis based on the curated TreeFam database (Family ID: TF323211) places UCP1 within the metazoan UCP subfamily of the SLC25 superfamily, showing the closest paralogous relationship to UCP2 and UCP3, and more distant evolutionary homology to SLC25A14 and SLC25A30 (Figure 1A) [18,19]. Multiple conserved segments were observed across species, particularly within the transmembrane regions and signature carrier motifs (Figure 1B). The evolutionary trajectory of UCP1, including its emergence, lineage-specific losses, and functional adaptations, has been comprehensively reviewed elsewhere, providing an important phylogenetic context for interpreting the regulatory and functional differences observed between rodents and humans [20].

The defining biochemical property of UCP1 is its ability to mediate proton conductance across the mitochondrial inner membrane. Under physiological conditions, UCP1 activity is activated by long-chain fatty acids and inhibited by purine nucleotides such as GDP, ADP, ATP, and GTP [2,21]. A broad range of cytosolic nucleotides may inhibit UCP1-mediated proton conductance under specific biochemical conditions, but the extent of inhibition depends on nucleotide species, concentration, membrane lipid composition, fatty acid availability, and experimental system.

Historically, both monomeric and dimeric models of UCP1 function have been proposed. More recent biochemical and structural evidence generally supports a monomeric functional unit, although earlier dimeric models contributed importantly to the development of the field. Whether transient oligomerization or membrane microdomain organization modulates UCP1 function in native mitochondria remains incompletely resolved.

### 2.2. High-Resolution Structures and Ligand-Binding Mechanisms

UCP1 activity is determined by the balance between fatty acid activation and nucleotide inhibition. Classical biochemical studies established that fatty acids are required for UCP1-mediated proton conductance, whereas nucleotides stabilize an inhibited state [2,21]. Mechanistically, fatty acids have been proposed either to act as proton-coupling cofactors or to favor conformational states permissive for proton leak; these models are not mutually exclusive.

Until recently, structural insight into UCP1 relied mainly on homology models based on the mitochondrial ADP/ATP carrier, which could not adequately explain ligand selectivity, pH dependence, or inhibition. This limitation was overcome in 2023, when two independent cryo-EM studies resolved human UCP1 in a GTP-bound inhibited conformation at near-atomic resolution, providing the first direct structural framework for ligand-dependent regulation [22,23,24]. In this inhibited conformation, UCP1 adopts a cytoplasmic-open state in which the central cavity is exposed to the intermembrane space while the matrix gate remains closed. GTP binds deeply within the cavity through an electrostatic network involving conserved basic residues, notably the arginine triad R84/R183/R277 together with K38 and K138. These interactions rigidify the transmembrane bundle and lock UCP1 in a non-conductive state.

These structures clarify several long-recognized biochemical features of nucleotide inhibition. Inhibitory potency appears to depend more strongly on phosphate charge than on nucleobase identity, consistent with the stronger effect of triphosphate nucleotides. Recent structural and functional data further suggest that pyrimidine nucleotides, including UTP and CTP, can also inhibit UCP1, extending the classical purine-centered model [25]. In addition, the pH dependence of inhibition has been linked to E191 in the central cavity: protonation of E191 at lower pH is predicted to reduce electrostatic repulsion and strengthen nucleotide binding, whereas deprotonation at higher pH weakens binding and facilitates activation [24].

Electrophysiological studies of brown fat mitochondria have shown that long-chain fatty acids activate UCP1-dependent proton currents, whereas nucleotides suppress them [21]. More recent molecular dynamics simulations and structure-guided mutagenesis suggest that fatty acid anions enter the central cavity from the intermembrane-space leaflet and engage a hydrophobic region that partially overlaps with the nucleotide-binding pocket. In this model, fatty acid binding destabilizes the inhibited state through steric and local conformational effects, potentially involving rearrangements of TM2 and TM4 that create a lateral hydrophobic exit pathway.

Within this updated framework, fatty acids are proposed to act not only as allosteric activators but also as mobile proton-coupling cofactors. A deprotonated fatty acid anion may engage the central cavity, become protonated via a coordinated hydrogen-bonding and water network involving conserved residues such as D28, then laterally repartition into the membrane and release its proton on the matrix side before re-entering a new cycle [26]. This model provides a mechanistic bridge between classical fatty acid cycling and cofactor-based mechanisms, although several steps remain to be validated directly.

The mitochondrial lipid environment also influences UCP1 function. Cardiolipin and phosphatidylethanolamine are enriched in the inner membrane and affect UCP1 stability, conformational flexibility, and ligand sensitivity. Cryo-EM structures identified three cardiolipin-binding sites per UCP1 monomer at matrix-side gaps between the three structural repeats. These bound cardiolipins stabilize the helical bundle and preserve the geometry of the ligand-binding cavity; depletion of cardiolipin causes irreversible misfolding and loss of proton transport activity. Cold-induced remodeling of cardiolipin acyl chains toward higher polyunsaturation may further lower the energetic barrier for conformational transitions and enhance responsiveness to fatty acid activators [27]. Accordingly, data from detergent-solubilized or reconstituted systems should be interpreted together with native mitochondrial assays.

Recent structural studies of mitochondrial carrier proteins have improved understanding of alternating-access mechanisms, gating networks, and ligand-binding pockets [16,17]. For UCP1, these advances establish a structural basis for nucleotide inhibition and provide a testable framework for fatty-acid-dependent activation. However, key questions remain unresolved, including the absence of a high-resolution activated matrix-open conformation, the limited direct evidence for the proposed fatty acid binding pose and lateral exit pathway, and the unclear structural basis of proton selectivity.

### 2.3. Tissue Distribution and Subcellular Localization

UCP1 is highly enriched in brown adipocytes and inducible beige adipocytes. In rodents, classical BAT is primarily located in interscapular and perirenal depots, whereas beige adipocytes emerge within white adipose tissue after cold exposure, β-adrenergic stimulation, exercise, hormonal cues, or pharmacological intervention [28,29,30,31]. In adult humans, metabolically active BAT is most consistently detected in supraclavicular and cervical depots by ^18^F-FDG PET/CT, although this signal reflects glucose uptake rather than UCP1 proton conductance directly [4,5,6,7,8].

Expression of UCP1 in non-adipose tissues—including kidney, liver, skeletal muscle, immune cells, and tumors—remains controversial. The main challenge is that UCP1 is highly abundant in brown adipocytes but typically present at extremely low or undetectable levels elsewhere, making detection vulnerable to technical artifacts. Major confounders include adipocyte contamination, antibody cross-reactivity with related carriers such as UCP2 and UCP3, discordance between low-abundance mRNA and functional protein expression, and ectopic reporter activity in Ucp1-Cre models [30,32,33]. Consistent with this, large-scale human proteomic resources generally show very low or undetectable UCP1 protein in most non-adipose tissues [33,34,35]. Although context-dependent expression in selected cell types cannot be fully excluded, such claims require stringent validation by orthogonal approaches, including cell-type-specific localization, mitochondrial fractionation, well-validated antibodies, proteomics, and functional bioenergetic assays [36,37,38]. Importantly, UCP1 mRNA alone does not establish thermogenic capacity.

At the subcellular level, UCP1 localizes to the mitochondrial inner membrane, where it modulates proton conductance and coupling efficiency [2,21,39]. Its functional impact depends not only on protein abundance but also on mitochondrial density, respiratory capacity, fatty acid supply, nucleotide inhibition, membrane lipid environment, and tissue oxygenation [15,40]. Accordingly, claims of functional UCP1 activity outside adipose tissue require evidence that the protein is mitochondrial, expressed at sufficient abundance, and associated with measurable changes in proton leak or respiration.

### 2.4. Transcriptional, Post-Transcriptional, and Post-Translational Regulation

UCP1 expression is regulated by a multilayered network involving sympathetic stimulation, nuclear receptors, transcriptional coactivators, chromatin remodeling, RNA metabolism, translation, and mitochondrial protein turnover.

**Figure 2:**
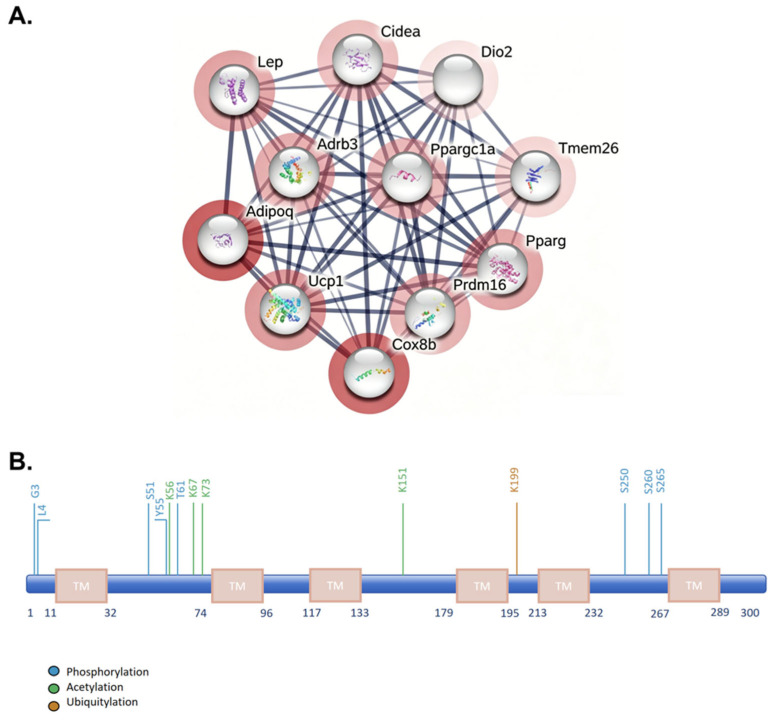
UCP1-centered protein association network and post-translational modification landscape. (**A**) Core protein association network centered on UCP1. Functionally related regulators of thermogenesis and adipocyte metabolism, including Adipoq, Ppargc1a, and Pparg, are shown. Node size and red color intensity represent the relative strength of predicted or curated associations (https://string-db.org/, accessed on 20 May 2026). (**B**) Schematic overview of post-translational modification (PTM) sites on UCP1, compiled from the PhosphoSitePlus database (https://www.phosphosite.org/homeAction, accessed on 20 May 2026). The blue bar represents the full-length UCP1 protein sequence, and pink rectangles indicate transmembrane (TM) domains. Vertical colored lines denote different modification types: blue, phosphorylation; green, acetylation; orange, ubiquitylation. Numbers indicate the corresponding amino acid positions of representative sites. Caution: This diagram includes both sites experimentally identified by high-throughput mass spectrometry and sites predicted by bioinformatic algorithms. The majority of displayed sites have not been functionally validated, and the presence of a modification site in this schematic does not imply confirmed physiological function or regulatory role.

Cold exposure activates sympathetic outflow to adipose tissue, leading to norepinephrine release and β-adrenergic receptor activation. The β3-adrenergic receptor β3-AR/cAMP/protein kinase A (PKA) cascade stimulates lipolysis, releases fatty acids that activate UCP1, and induces transcriptional programs that increase UCP1 abundance [1,3,28]. Transcriptional activation involves cAMP-response element-binding protein, peroxisome proliferator-activated receptors (PPARs), PPARγ coactivator 1α (PGC1α), PR domain-containing protein 16 (PRDM16), early B-cell factor 2 (EBF2), and thyroid hormone signaling through type II iodothyronine deiodinase (DIO2) [29,30,31,41,42] (Figure 2A).

At the post-transcriptional level, UCP1 regulation is increasingly recognized as a critical determinant of adipocyte thermogenic competence. In addition to RNA-binding proteins, a growing number of microRNAs (miRNAs) have been implicated in the control of brown/beige adipocyte differentiation and UCP1 expression. Among these, miR-133 and miR-155 are generally considered anti-thermogenic regulators that suppress brown/beige adipocyte programming by targeting key upstream factors such as PRDM16 and related transcriptional networks, whereas miR-196a promotes beige adipocyte recruitment and thermogenic gene induction in several experimental models [22,43,44,45]. Other miRNAs, including the miR-27 family, miR-34a, and circulating miR-92a, have also been linked to adipocyte browning, BAT activity, or UCP1-associated thermogenic status. Importantly, post-transcriptional thermogenic regulation is not restricted to cell-intrinsic miRNA programs. Emerging evidence indicates that extracellular vesicles (EVs) and exosomes can transfer miRNAs and other regulatory cargos between adipocytes, immune cells, skeletal muscle, and other tissues, thereby modulating adipose inflammation, browning capacity, and UCP1 expression in a non-cell-autonomous manner. Although many of these pathways remain incompletely defined, EV/exosome-mediated signaling is increasingly viewed as an important regulatory layer connecting thermogenic adipose tissue to systemic metabolic communication [22,44,45,46,47].

Post-translational modifications (PTMs) of UCP1 remain incompletely characterized, and the functional relevance of most reported sites remains unvalidated. As summarized in Figure 2B, currently available UCP1 PTM data can be stratified into three evidence levels:

Experimentally detected sites (high-throughput identification): A subset of phosphorylation, acetylation, and ubiquitylation sites have been identified by untargeted mass spectrometry-based proteomic screening, primarily in mouse brown adipose tissue or cultured adipocyte models. Representative detected residues include phosphorylation sites (e.g., Ser3, Ser35, Thr133, Ser239), acetylation sites (e.g., Lys117, Lys179, Lys267), and ubiquitylation sites (e.g., Lys23, Lys117, Lys195, Lys289). Nearly all of these MS-identified sites were discovered in untargeted discovery studies, and their physiological regulation and functional impacts on UCP1 activity remain largely uncharacterized [48].

Bioinformatically predicted sites: Additional putative modification residues have been inferred in silico from sequence motif analysis and structural prediction algorithms, including candidate phosphorylation, succinylation, and sumoylation sites. These predictions serve as hypotheses for further investigation and require independent experimental validation.

Functionally validated sites: Only a very small number of UCP1 PTMs have received targeted functional investigation. Early biochemical studies suggested that PKA-mediated phosphorylation modulates UCP1 nucleotide sensitivity, but the exact modified residues and quantitative effects on proton conductance have not been definitively mapped. Ubiquitylation of UCP1 has been implicated in protein turnover and degradation under thermoneutral conditions, but the specific lysine residues and regulatory E3 ligases remain incompletely defined.

Notably, species-specific differences in UCP1 PTM profiles between rodents and humans have not been systematically evaluated, and most available data are derived from mouse studies. Overall, the vast majority of reported UCP1 PTM sites lack functional confirmation; their effects on UCP1 protein stability, nucleotide binding affinity, fatty acid activation efficiency, and proton transport activity therefore remain important open questions requiring targeted biochemical and mutational validation.

## 3. Biological Functions of UCP1 in Cellular and Systemic Homeostasis

### 3.1. Adaptive Thermogenesis and Body Temperature Regulation

UCP1-dependent thermogenesis is the best-characterized function of brown and beige adipocytes. During cold exposure, sympathetic activation promotes lipolysis and releases fatty acids. These fatty acids serve two complementary roles: they provide substrates for β-oxidation and directly activate UCP1-mediated proton conductance. As protons re-enter the mitochondrial matrix independently of ATP synthase, mitochondrial respiration accelerates and chemical energy is released as heat [1,2,3,21].

Genetic studies in mice demonstrate the physiological importance of UCP1. UCP1-deficient mice are cold-sensitive and unable to mount normal non-shivering thermogenesis under acute cold challenge [49]. However, their obesity phenotype depends strongly on housing temperature. At standard laboratory temperatures, mice experience chronic mild cold stress, which activates compensatory mechanisms. Under thermoneutral conditions, loss of UCP1 more clearly impairs diet-induced thermogenesis and predisposes to obesity [50].

**Figure 3:**
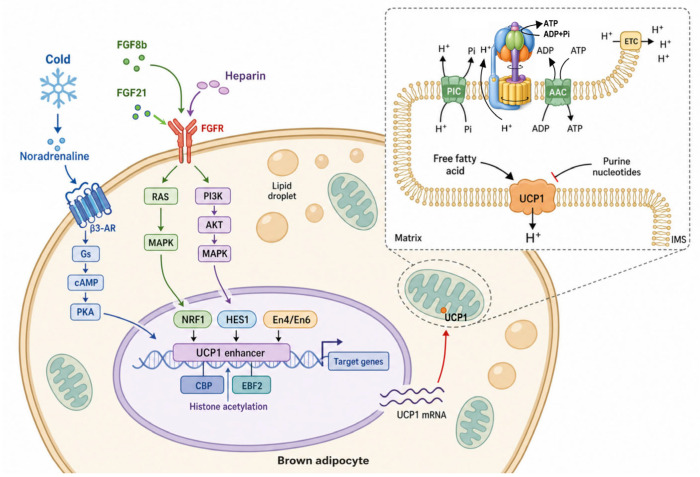
Mechanistic model illustrating the regulation of brown-adipocyte thermogenesis and UCP1 activity by cold stimulation and FGF signaling. Cold exposure activates the beta3-adrenergic receptor (beta3-AR)-cAMP-protein kinase A (PKA) pathway, whereas FGF8b/FGF21-mediated FGF receptor signaling activates RAS-MAPK and PI3K-AKT-MAPK cascades. These signaling pathways converge on enhancer and transcriptional regulatory programs, including histone acetylation at UCP1 enhancers (En4/En6) and transcriptional regulators such as NRF1 and HES1, thereby promoting UCP1 mRNA expression. At the mitochondrial level, UCP1 localized in the inner mitochondrial membrane mediates fatty-acid-activated proton leak, uncouples oxidative phosphorylation from ATP synthesis, and increases thermogenic energy expenditure. In contrast, purine nucleotides inhibit UCP1 activity by binding to the nucleotide-binding pocket.

Recent studies have refined the regulatory hierarchy of adipose thermogenesis by identifying mechanisms that either directly modulate UCP1 activity or operate in parallel as UCP1-independent compensatory pathways. These include creatine-driven substrate cycling, calcium cycling involving SERCA2b, triglyceride/fatty acid cycling, and futile metabolic cycles in beige adipocytes [13,14,15]. These pathways are important because BAT activity and whole-body energy expenditure cannot be attributed exclusively to UCP1 abundance. Together, these regulatory mechanisms provide a framework for understanding how extracellular cold and growth factor signals are translated into UCP1 transcriptional activation and mitochondrial thermogenic output (Figure 3).

### 3.2. Lipid Metabolism, Glucose Utilization, and Systemic Energy Balance

Although UCP2 and UCP3 also participate in mitochondrial substrate handling and oxidative stress responses, this review focuses on UCP1 because of its unique thermogenic function and strong enrichment in brown/beige adipocytes.

#### 3.2.1. UCP1 and Lipid Mobilization/Fatty Acid Oxidation

UCP1 activation increases the demand for mitochondrial oxidation. Brown and beige adipocytes therefore consume intracellular triglycerides and circulating fatty acids, including triglyceride-rich lipoprotein-derived fatty acids. BAT activation can accelerate plasma triglyceride clearance and improve lipid profiles in preclinical models [51]. In humans, cold exposure increases BAT oxidative metabolism and can enhance uptake of glucose and fatty acids into BAT depots [52,53,54].

Because fatty acids are both fuels and activators of UCP1, lipid metabolism is tightly coupled to thermogenesis. However, excessive lipid flux may induce lipotoxicity if mitochondrial oxidative capacity is insufficient. Therefore, effective UCP1-mediated lipid disposal requires coordinated induction of mitochondrial biogenesis, respiratory-chain capacity, vascularization, and adipocyte differentiation.

#### 3.2.2. UCP1 and Glucose Utilization/Insulin Sensitivity

Activated BAT consumes glucose, and human BAT glucose uptake is readily detected by ^18^F-FDG PET/CT after cold exposure [4,5,6,7,8]. Cold acclimation can improve insulin sensitivity in humans, although the relative contributions of BAT, skeletal muscle, and systemic endocrine adaptation vary among studies [55]. In individuals with metabolically active BAT, lower adiposity, improved glucose homeostasis, and favorable cardiometabolic profiles have been reported [7,8].

Importantly, ^18^F-FDG uptake reflects glucose utilization, not UCP1-mediated proton conductance. This distinction is critical, as BAT imaging signals are therefore indirect markers of thermogenic activity rather than direct measures of UCP1 function. To address this limitation, Porter systematically reviewed methods for direct biochemical quantification of UCP1-dependent proton conductance in human brown adipose tissue biopsies, detailing approaches to assess mitochondrial bioenergetics and uncoupling activity and offering a methodological framework that complements imaging-based readouts [56]. A more comprehensive evaluation of UCP1-related metabolic activity may be achieved by integrating multimodal techniques, including PET tracers, MRI, infrared thermography, tissue biopsies, and circulating biomarkers.

### 3.3. Redox Homeostasis and Mitochondrial ROS Control

Mitochondrial reactive oxygen species (ROS) are generated primarily at respiratory-chain complexes I and III. Mild mitochondrial uncoupling can lower mitochondrial membrane potential and reduce electron leak, thereby limiting ROS production under certain conditions. UCP1-mediated proton leak may therefore contribute to redox homeostasis in brown and beige adipocytes [11,12].

The relationship between UCP1 and ROS is bidirectional. On one hand, UCP1 activation can decrease mitochondrial hyperpolarization and reduce ROS generation. On the other hand, ROS and lipid peroxidation products may modulate thermogenic signaling and mitochondrial function. These findings support the concept that UCP1 may function as an important interface between mitochondrial bioenergetics, redox regulation, and cell survival, particularly in metabolically active tissues.

However, UCP1 should not be viewed as a universal antioxidant protein. Its redox effects depend on tissue context, mitochondrial substrate supply, oxygen availability, respiratory-chain capacity, and the presence of compensatory antioxidant systems. Direct evidence for UCP1-mediated ROS regulation is strongest in brown/beige adipocytes and selected experimental systems; evidence in non-adipose tissues remains more limited.

### 3.4. Inflammation, Immune Remodeling, and Interorgan Communication

The immunomodulatory effects of UCP1 can be divided into two categories: (1) systemic and paracrine effects driven by UCP1-expressing adipocytes, including BAT-derived endocrine signals, metabolite clearance, and adipose immune remodeling that influence distant organs; (2) potential cell-autonomous effects in rare non-adipose cell types where UCP1 may be induced under specific conditions. The vast majority of immunomodulatory actions of UCP1 are mediated by the adipose tissue compartment.

BAT and beige adipose tissue communicate with immune and metabolic organs through secreted factors, lipids, metabolites, and extracellular vesicles. Cold exposure and β-adrenergic activation can reshape adipose immune cell composition, promote alternatively activated macrophage phenotypes in some contexts, and alter systemic inflammatory tone [57,58]. Conversely, chronic obesity-associated inflammation impairs adipocyte thermogenic competence and suppresses UCP1 expression.

BAT also participates in interorgan metabolite handling. For example, succinate has been identified as a circulating metabolite that can accumulate in BAT during cold exposure and support thermogenesis [59]. Such findings suggest that UCP1-expressing adipocytes may influence systemic metabolism not only by burning substrates but also by modifying metabolite availability and inflammatory signaling.

In addition, EVs, including exosomes, are emerging as important mediators of adipose–organ communication relevant to UCP1 regulation. Thermogenic adipocytes can both secrete and respond to EV-associated cargos, including miRNAs, lncRNAs, proteins, and lipids, which influence adipocyte differentiation, inflammatory tone, mitochondrial function, and browning capacity. EV-mediated transfer of thermogenesis-related miRNAs provides a plausible mechanism by which distant tissues such as skeletal muscle, liver, immune cells, or other adipose depots may modulate UCP1 expression in a non-cell-autonomous manner. Conversely, obesity- or inflammation-associated EVs may suppress thermogenic programming and contribute to BAT dysfunction. Although many of these pathways remain incompletely resolved, EV/exosome biology is increasingly recognized as a key layer of systemic regulation linking adipose thermogenesis to interorgan metabolic communication [60,61].

## 4. UCP1 in Human Diseases

To avoid overinterpretation, disease-related evidence is categorized in this section according to its strength and directness. Strong/adipose-autonomous evidence refers to direct UCP1 function demonstrated in brown or beige adipocytes by genetic, mitochondrial, or thermogenic assays. Indirect adipose–organ crosstalk refers to disease effects mediated by UCP1-positive thermogenic adipose tissue through endocrine signaling, metabolite handling, lipid and glucose clearance, inflammatory remodeling, or interorgan communication. Animal-model evidence refers to findings mainly derived from rodent or cell-based experimental systems without sufficient human validation. Associative human evidence refers to imaging, genetic, transcriptomic, clinical, or epidemiological associations that do not directly establish causal UCP1 function. Speculative or incompletely validated evidence refers to claims of local UCP1 expression or function in non-adipose tissues that require further confirmation by cell-type-specific protein detection, mitochondrial localization, antibody specificity controls, and functional proton conductance assays. This framework is used throughout the following disease sections to distinguish direct adipocyte-autonomous UCP1 actions from indirect systemic effects and from preliminary local non-adipose UCP1 findings.

### 4.1. Obesity and T2DM

Evidence level: strong adipose-autonomous evidence and associative human evidence. In obesity and T2DM, the strongest evidence supports UCP1 as an adipocyte-autonomous thermogenic effector in brown and beige adipocytes, whereas human studies mainly provide associative evidence linking active brown adipose tissue with improved adiposity, glucose homeostasis, lipid metabolism, and cardiometabolic profiles.

UCP1-positive brown and beige adipose tissues are metabolically active organs involved in energy expenditure, substrate clearance, endocrine signaling, vascular remodeling, and immune–adipocyte interactions [62,63]. These functions are highly relevant to obesity and T2DM, which are characterized by adipose dysfunction, inflammation, insulin resistance, and impaired nutrient handling [64].

Experimental studies show that activation or expansion of brown/beige adipocytes improves glucose tolerance, fatty acid oxidation, triglyceride-rich lipoprotein clearance, and energy expenditure. Conversely, impaired thermogenic identity or UCP1 dysfunction can worsen diet-induced metabolic abnormalities, particularly under thermoneutral conditions. However, thermogenesis is not exclusively UCP1-dependent, as creatine cycling, calcium cycling, and other futile metabolic pathways may also contribute [62,65].

Human data support an association between active brown adipose tissue (BAT) and better cardiometabolic health. Large PET/CT studies show that detectable BAT is linked to lower prevalence of T2DM, dyslipidemia, coronary artery disease, hypertension, and heart failure [8]. BAT activation can enhance glucose and lipid uptake during cold exposure, although its overall contribution to daily energy expenditure is variable and often reduced in obesity and T2DM. Beyond adult cross-sectional studies, emerging evidence in pediatric populations suggests that reduced UCP1 expression in subcutaneous adipose tissue may serve as an early predictor of childhood obesity, highlighting the potential utility of UCP1-based biomarkers across different life stages [66].

A major limitation is that most clinical studies use ^18^F-FDG PET/CT, which measures glucose uptake rather than total oxidative metabolism. Since BAT also uses fatty acids and intracellular triglycerides, FDG-based imaging may underestimate BAT activity, especially in insulin-resistant individuals. Newer PET tracers, MRI-based methods, infrared thermography, and multimodal approaches are therefore needed [63].

Interventions such as repeated mild cold exposure can recruit BAT and improve insulin sensitivity in some subjects, but clinical use is limited by adherence and variability. Pharmacological activation with β-adrenergic agonists, including the β3-AR agonist mirabegron, can increase human BAT activity and may improve insulin sensitivity or lipid profiles in selected settings. However, human BAT adrenergic regulation does not fully mirror the β3-AR-dominant paradigm established in mice, and high-dose pharmacological stimulation may produce cardiovascular sympathetic effects. Therefore, safer translational strategies should consider human-specific β2/β3-adrenergic signaling, dose–response relationships, and patient-level variability rather than relying solely on rodent β3-AR models [67]. Safer strategies should aim to enhance BAT recruitment, mitochondrial function, or downstream thermogenic pathways without generalized adrenergic stimulation.

Single-cell studies reveal that human thermogenic adipose tissue is heterogeneous and influenced by depot, obesity, and insulin resistance [68,69]. Obesity-associated hypertrophy, inflammation, fibrosis, vascular rarefaction, mitochondrial stress, and altered sympathetic input may suppress UCP1 expression and thermogenic capacity. Thus, effective therapy may require restoring the broader adipose microenvironment, not only increasing UCP1.

BAT and beige fat may also protect metabolism by acting as sinks for glucose, fatty acids, and triglyceride-rich lipoproteins, reducing nutrient overload in liver, muscle, and pancreatic β-cells. In addition, thermogenic adipocytes secrete batokines that influence hepatic metabolism, vascular function, immune tone, appetite, and muscle substrate use [62,70].

Genetic evidence linking UCP1 variants to obesity or T2DM is inconsistent. The common-3826A/G polymorphism has been associated with metabolic traits in some studies, but effects are small and population-dependent. Large genetic studies indicate that obesity and T2DM are highly polygenic [71,72], suggesting that UCP1 variation is more likely a modifier of thermogenic capacity than a major disease determinant.

Overall, targeting UCP1-positive thermogenic adipose tissue is biologically plausible for obesity and T2DM, but translation remains challenging. Human BAT is limited, heterogeneous, and affected by age, sex, adiposity, temperature, season, sympathetic tone, medications, and metabolic status. Future studies should integrate advanced imaging, tissue UCP1 and mitochondrial assessment, single-cell profiling, endocrine analyses, genetics, and deep metabolic phenotyping to determine whether BAT activation can become a safe and clinically meaningful therapy.

### 4.2. Cardiovascular Diseases and Heart Failure

Evidence level: indirect adipose–cardiovascular crosstalk, supported by animal-model and associative human evidence. It must be explicitly clarified that endogenous UCP1 expression in cardiomyocytes is absent or negligible under physiological conditions in both humans and common laboratory rodents. All cardiovascular regulatory effects attributed to UCP1 are primarily mediated by UCP1-expressing thermogenic adipose depots—including epicardial adipose tissue, perivascular adipose tissue, and systemic brown/beige adipose tissue—rather than by cardiomyocyte-intrinsic UCP1 protein [73,74]. UCP1-positive brown, beige, epicardial, and perivascular adipocytes may affect the heart and vessels by regulating fatty acid uptake, lipid oxidation, heat production, inflammation, paracrine signaling, and substrate supply.

Epicardial adipose tissue is especially important because it directly contacts the myocardium and coronary arteries. Human epicardial fat expresses UCP1 and other brown/beige adipocyte markers, indicating a thermogenic and oxidative phenotype [75,76]. This UCP1 expression is localized exclusively to adipocytes within the epicardial fat depot, not to underlying cardiomyocytes. Under physiological conditions, epicardial adipose UCP1 activity may help buffer excess local fatty acids and maintain a favorable metabolic environment for the heart and coronary vessels. However, in coronary artery disease, epicardial fat often becomes enlarged, inflamed, and fibrotic, which may override any protective UCP1-related effects and promote endothelial dysfunction, coronary inflammation, and plaque progression.

The role of UCP1 in atherosclerosis is context-dependent. Activation of brown fat can enhance lipid clearance and reduce atherosclerosis in experimental models [77]. In contrast, excessive cold-induced thermogenesis may increase lipolysis and lipid flux, thereby promoting plaque growth and instability under pro-atherogenic conditions [78]. Thus, UCP1-mediated thermogenesis may be beneficial when lipid mobilization is matched by oxidation, but harmful when lipolysis exceeds lipid clearance capacity.

In heart failure, UCP1 is linked to cardiac–adipose endocrine communication. Elevated atrial and B-type natriuretic peptides can induce adipocyte browning and UCP1 expression through cGMP–p38 MAPK signaling [79,80]. This response may initially support lipid utilization, but persistent activation in advanced heart failure may contribute to energy wasting, adipose loss, and cardiac cachexia.

With regard to ischemia–reperfusion injury, it is critical to distinguish physiological endogenous expression from artificial transgenic manipulation. Although endogenous UCP1 expression in cardiomyocytes is minimal, transgenic cardiac UCP1 expression can reduce ischemia–reperfusion injury, likely by lowering mitochondrial membrane potential and oxidative stress [80]. However, excessive uncoupling could impair ATP production, so such models should be viewed as mechanistic rather than physiological evidence.

Overall, UCP1 has both protective and detrimental potential in cardiovascular disease. Its effects depend on adipose depot, disease stage, lipid availability, inflammation, sympathetic activity, natriuretic peptide signaling, and oxidative capacity. In this context, UCP1 is most relevant to epicardial adipose function, lipid flux, coronary microenvironment regulation, heart–adipose signaling, and mitochondrial stress responses.

### 4.3. Acute Kidney Injury and Renal Fibrosis

Evidence level: animal-model evidence, indirect adipose–kidney crosstalk, and speculative local renal evidence. In kidney injury and renal fibrosis, reported UCP1-related protective effects are mainly derived from preclinical models and systemic metabolic studies. Direct local renal UCP1 protein expression and functional activity remain incompletely validated and should be interpreted cautiously. The kidney is a highly energy-demanding organ, and mitochondrial dysfunction, oxidative stress, inflammation, and fibrotic remodeling are central events in acute kidney injury (AKI), chronic kidney disease (CKD) progression, diabetic kidney disease, and sepsis-associated renal injury. Uremia-related metabolic stress can further amplify oxidative injury; for example, urea-induced reactive oxygen species (ROS) have been shown to cause endothelial dysfunction in chronic renal failure [81,82,83]. Although UCP1 is classically recognized as a thermogenic protein in brown and beige adipocytes, emerging evidence suggests that UCP1-related pathways may confer renal protection by modulating mitochondrial redox homeostasis, inflammatory responses, and fibrosis-associated signaling [84].

It is critical to distinguish two distinct pathways by which UCP1 may influence renal outcomes: indirect systemic effects mediated by adipose tissue UCP1, and direct effects of locally expressed UCP1 in renal cells. The latter remains highly controversial and has not been universally verified. A recent rigorous study using multiple independent methods concluded that there is no detectable endogenous UCP1 protein in adult mouse or human kidney parenchyma, and that earlier positive signals may be explained by perirenal adipose tissue contamination, infiltrating immune cells, or non-specific antibody reactivity [33]. Ucp1-Cre reporter signals observed in kidney also do not reliably reflect endogenous UCP1 protein expression, as they may trace transient developmental promoter activity or ectopic Cre expression [29]. Thus, most renoprotective effects attributed to UCP1 are more likely mediated systemically by brown/beige adipose tissue, rather than by UCP1 intrinsically expressed in renal tubular or glomerular cells.

In experimental AKI models, pharmacological interventions that modulate UCP1-associated pathways have been reported to correlate with reduced mitochondrial ROS levels and attenuated tubular epithelial injury. However, these observations are largely derived from whole-body pharmacological studies, and it remains unresolved whether the protective effects are driven by local renal UCP1 or by systemic metabolic and redox improvements secondary to adipose UCP1 activation. It has been proposed that mild mitochondrial uncoupling could lower excessive mitochondrial membrane potential, limit electron leakage from the respiratory chain, and thereby reduce tubular injury [84]. This putative mechanism has been discussed in the context of ischemia–reperfusion injury, nephrotoxicity, and sepsis-associated AKI, where mitochondrial ROS amplification is a well-established driver of tubular damage and inflammatory activation. In sepsis models, pharmacological activation of the PPAR-γ/UCP1 axis (e.g., by baicalin administration) has been associated with improved mitochondrial function and suppressed inflammatory responses, leading to the hypothesis of a metabolic–mitochondrial–anti-inflammatory protective network [85].

Several preclinical studies have suggested a possible association between UCP1-related pathways and renal fibrogenesis, but the causal role and cellular source of UCP1 in these processes remain unconfirmed. In rodent models of renal interstitial fibrosis, UCP1 upregulation was reported to be associated with attenuated fibrotic progression, which has been proposed to occur via preservation of SIRT3 stability and reduction in oxidative stress [86]. Because SIRT3 regulates mitochondrial antioxidant enzymes and metabolic homeostasis, the UCP1/SIRT3 axis may indirectly suppress profibrotic pathways such as TGF-β/Smad signaling, extracellular matrix deposition, and myofibroblast activation. In chronic renal failure models, UCP1-related regulation has further been associated with PHD2, RIPK3/AKT/TGF-β signaling, hypoxia adaptation, necroptosis, and fibrotic remodeling [87]. In diabetic kidney disease, ANXA1/UCP1 signaling has been proposed to maintain mitochondrial homeostasis and reduce high-glucose-induced oxidative injury [88].

Importantly, the role of UCP1 in kidney disease appears to be context- and tissue-dependent. Local or cell-associated UCP1 activation may protect renal cells by limiting mitochondrial ROS and inflammatory injury, whereas excessive adipose UCP1 activation in advanced CKD may contribute to pathological energy expenditure, adipose browning, cachexia, and muscle wasting [89,90]. Growth hormone has been reported to improve adipose tissue browning and muscle wasting in CKD-associated cachexia, supporting the metabolic relevance of adipose thermogenic remodeling in CKD [89]. Similarly, parathyroid hormone-induced adipose tissue browning has been proposed as a pathway contributing to muscle wasting, further highlighting the complex endocrine links among CKD, adipose metabolism, and systemic energy imbalance [90]. In addition, kidney-related systemic complications may involve broader inter-organ metabolic communication, as suggested by studies on irisin-activated osteoblasts in renal osteodystrophy and brown adipose tissue-derived protective factors in ischemic injury models [91,92].

Despite these promising observations, direct renal UCP1 function remains incompletely defined. UCP1 expression in kidney tissue is generally low and technically challenging to validate, making it difficult to distinguish true local renal effects from adipose-derived endocrine actions or systemic metabolic improvement. Future studies should therefore integrate kidney cell-specific genetic models, adipose-specific UCP1 manipulation, rigorously validated antibodies, mitochondrial functional assays, and single-cell or spatial profiling approaches. Such strategies will be essential to clarify whether UCP1 is a direct renal effector or primarily a mediator of adipose–kidney metabolic crosstalk in AKI and renal fibrosis.

### 4.4. Neurological Disorders and Neuroprotection

Evidence level: indirect adipose–brain crosstalk and associative/preclinical evidence. UCP1 is a classical brown/beige adipocyte mitochondrial protein, and there is no universally verified, functionally relevant endogenous UCP1 expression in neurons or glial cells of adult humans or common laboratory rodents. Large-scale proteomic and transcriptomic atlases show only trace or undetectable UCP1 levels in the central nervous system (CNS), and brain mitochondrial uncoupling is primarily mediated by the paralogues UCP2, UCP4, and UCP5, which are broadly expressed in neural cells and have well-documented roles in redox regulation and neuroprotection [8,93]. Neuronal UCP1 expression has been reported exclusively in hibernating species such as the thirteen-lined ground squirrel, where it supports local thermogenesis during torpor as a specialized evolutionary adaptation; this pattern is not conserved in non-hibernating mammals.

Therefore, BAT/UCP1 influences neurological disorders indirectly through systemic metabolic, endocrine, inflammatory, and redox pathways, rather than via local UCP1 expression in neural tissue. Human epidemiological evidence indicates that active BAT is associated with better cardiometabolic health, improved glucose and lipid metabolism, and lower inflammatory burden, all of which are relevant to brain aging and neurodegeneration. Since obesity, diabetes, vascular risk factors, insulin resistance, and chronic inflammation are linked to cognitive decline and dementia, BAT/UCP1 activation may theoretically reduce peripheral drivers of neuroinflammation and neuronal stress [94,95,96].

Thermogenic adipose tissue may also affect the brain through endocrine crosstalk. BAT-derived or BAT-associated mediators, including FGF21 and other BATokines, can regulate energy balance, sympathetic activity, circadian behavior, and systemic metabolism [93,97,98]. Exercise- and metabolism-related factors such as FNDC5/irisin have also been implicated in brain function; experimental studies show that irisin can improve synaptic plasticity and memory in Alzheimer’s disease models, and newer work supports its role in cognitive regulation [99,100]. These findings suggest that BAT/UCP1-related metabolic activation may contribute to neuroprotection indirectly by improving whole-body metabolic homeostasis and adipose–brain communication.

Compared with UCP1, brain-expressed uncoupling proteins such as UCP2, UCP4, and UCP5 have stronger evidence in neuronal redox control, mitochondrial stress resistance, and injury protection. For example, UCP2 has been shown to reduce neuronal death and improve outcomes after stroke or brain trauma [101]. Therefore, in neurological disorders, UCP1 is best interpreted as a peripheral regulator of neuro-metabolic homeostasis rather than a direct CNS effector.

### 4.5. Liver Disease, Steatosis, and Fibrosis

Evidence level: indirect adipose–liver crosstalk, animal-model evidence, and limited associative human evidence. In liver disease, UCP1-related effects are most plausibly mediated by thermogenic adipose tissue through systemic lipid partitioning, glucose disposal, adipokine secretion, metabolite handling, and inflammatory regulation, whereas direct hepatic UCP1 function remains insufficiently established. Non-alcoholic fatty liver disease (NAFLD), now increasingly termed metabolic dysfunction-associated steatotic liver disease (MASLD), is strongly linked to obesity, insulin resistance, dyslipidemia, and chronic inflammation [102,103]. UCP1-expressing adipose tissue may influence liver disease through systemic substrate disposal, adipokine secretion, metabolite clearance, and inflammatory regulation.

BAT activation can increase fatty acid oxidation and glucose disposal, thereby reducing substrate overflow to the liver. In experimental models, beige adipocyte induction has been associated with improved hepatic steatosis and insulin sensitivity. Succinate metabolism provides another potential connection between thermogenic adipose tissue and liver inflammation. Succinate can act as both a mitochondrial metabolite and extracellular signaling molecule through succinate receptor 1 (SUCNR1). BAT uptake and metabolism of succinate during thermogenic activation may influence circulating succinate levels and inflammatory signaling [59]. However, the extent to which UCP1-dependent succinate handling directly modifies human liver fibrosis remains to be established.

Therapeutically, strategies that activate BAT/UCP1 may benefit fatty liver disease by improving whole-body energy balance and lipid partitioning. Yet direct clinical evidence remains limited, and endpoints such as liver fat content, fibrosis stage, inflammatory markers, and long-term outcomes must be evaluated in controlled human studies.

### 4.6. Cancer Metabolism and Tumor Microenvironment

Evidence level: relatively strong adipose-tissue evidence for cancer cachexia, exploratory transcriptomic evidence for tumor-associated UCP1 expression, and preliminary correlative evidence for tumor immune microenvironment associations. UCP1 participates in cancer biology through two conceptually distinct pathways: systemic indirect effects mediated by UCP1-expressing adipose tissue, and putative direct effects of endogenous UCP1 expressed within tumor or immune cells. The former is well supported by extensive preclinical and clinical evidence, whereas the latter remains heterogeneous, incompletely validated, and not universally accepted. It is essential to distinguish these two levels of action, as they have different mechanistic bases and therapeutic implications [104,105,106].

#### 4.6.1. Systemic Effects of Adipose Tissue UCP1 in Cancer: Cachexia and Metabolic Crosstalk

One well-established cancer-related context is cachexia. Tumor-derived and host-derived factors can promote adipose tissue remodeling, white adipose tissue browning, and increased expression of thermogenic genes, including *UCP1*, thereby contributing to elevated energy expenditure, lipid mobilization, and progressive weight loss. For example, tumor-derived parathyroid hormone-related protein has been shown to drive adipose browning and cancer cachexia in experimental models [107]. Similarly, activation of brown adipose tissue and browning of white adipose depots have been observed in cachexia-associated metabolic wasting [108]. More recent studies in pancreatic cancer further indicate that adipose remodeling in cachexia is regulated by multiple tumor–host communication pathways. Lipocalin-2 has been reported to mediate adipocyte–muscle–tumor communication and systemic thermoregulatory disturbances in pancreatic cancer cachexia, suggesting that adipose-derived metabolic signals may contribute not only to wasting but also to altered body temperature control [109]. In pancreatic ductal adenocarcinoma, activin A signaling has also been implicated in visceral adipose tissue remodeling during cachexia, supporting the concept that cytokine- and growth factor-driven adipose reprogramming is a central component of cancer-associated metabolic decline [110]. These findings underscore the potential risk associated with uncontrolled or sustained UCP1-related thermogenic activation in patients with advanced cancer, particularly in tumor types prone to cachexia. Nevertheless, cancer cachexia should not be interpreted simply as beneficial thermogenesis; rather, it represents a maladaptive systemic metabolic syndrome in which UCP1 induction may act in concert with inflammation, lipolysis, muscle wasting, and endocrine dysregulation.

Beyond cachexia, adipose tissue UCP1 activity may influence tumor progression indirectly by modulating systemic metabolic state, circulating lipid and glucose levels, adipokine secretion, and systemic immune tone. Thermogenic adipose tissue remodeling affects whole-body nutrient partitioning, insulin sensitivity, and inflammatory status, all of which shape the tumor macroenvironment and disease trajectory.

#### 4.6.2. Endogenous UCP1 in Tumor Cells: Evidence and Limitations

Reports of UCP1 expression within tumor cells have appeared in the literature, but their interpretation requires caution, and the findings have not been universally verified across independent laboratories.

**Figure 4:**
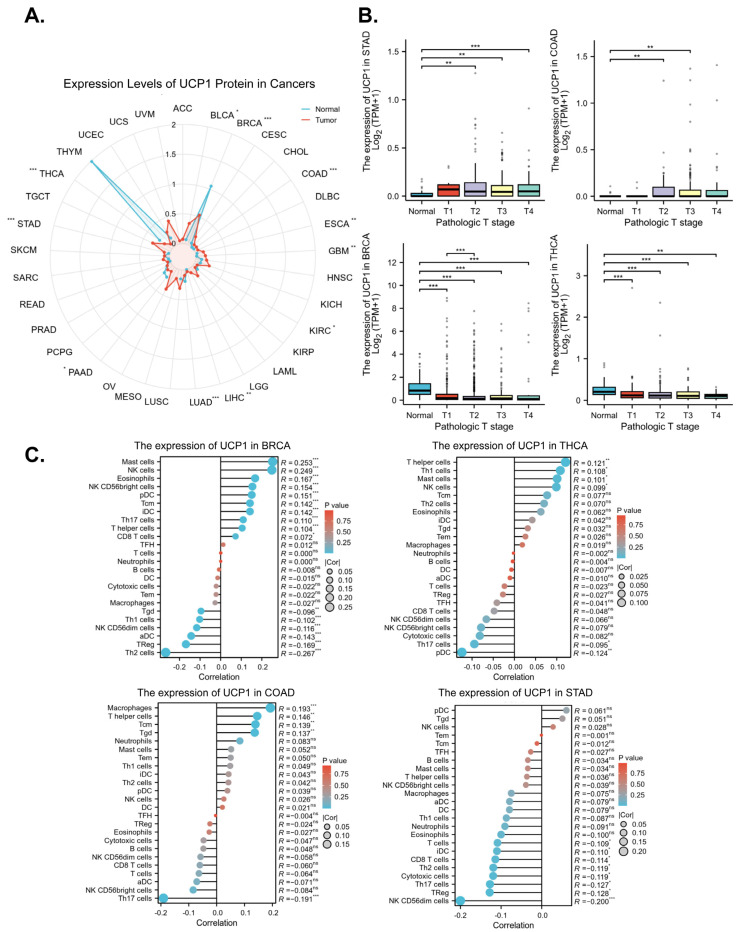
Pan-cancer expression profile of UCP1 and its associations with pathological stage and immune cell infiltration. (**A**) Radar plot showing UCP1 expression levels across 33 human cancer types and corresponding normal tissues based on The Cancer Genome Atlas (TCGA) database (https://portal.gdc.cancer.gov, accessed on 26 May 2026). Blue indicates normal tissues, red indicates tumor tissues, and radial-axis values represent relative UCP1 expression levels after log_2_(TPM + 1) transformation. Differential expression between tumor and normal groups was tested by the Mann–Whitney U test (Wilcoxon rank-sum test). *** p < 0.001, ** p < 0.01, and * p < 0.05. (**B**) Box plots showing the association between UCP1 expression and pathological T stage in stomach adenocarcinoma (STAD), colon adenocarcinoma (COAD), breast invasive carcinoma (BRCA), and thyroid carcinoma (THCA). The *y*-axis represents normalized UCP1 expression [log2(TPM + 1)], and the *x*-axis shows normal controls and tumor stages T1–T4. Between-group differences were assessed by the Kruskal–Wallis test, followed by Dunn’s post hoc test for pairwise comparisons. *** p < 0.001 and ** p < 0.01. (**C**) Lollipop plot showing Spearman correlations between UCP1 expression and the relative abundance of 24 immune cell types in the tumor microenvironment of STAD, COAD, BRCA, and THCA. Immune cell infiltration levels were estimated by the ssGSEA algorithm based on validated immune gene signatures. The *x*-axis represents the correlation coefficient; positive and negative values indicate positive and inverse correlations, respectively. Point color denotes the *p*-value of the correlation test, and point size represents the absolute correlation coefficient. Note: Data processing and visualization were performed on the HelixLife platform (www.helixlife.cn, accessed on 26 May 2026).

To systematically investigate the expression landscape and clinical relevance of UCP1 in human cancers, we performed an integrative bioinformatics analysis on the transcriptomic data of 33 cancer types from The Cancer Genome Atlas (TCGA) using the HelixLife online platform (www.helixlife.cn, accessed on 25 May 2026) (Tables S1 and S2). UCP1 exhibited a highly tissue-restricted expression pattern in normal tissues, with detectable expression mainly in thyroid and gastric tissues, while most other normal tissues showed very low or undetectable levels. In tumor tissues, UCP1 was generally expressed at low levels and was frequently downregulated compared with corresponding normal tissues. Among the analyzed cancer types, stomach adenocarcinoma (STAD), colon adenocarcinoma (COAD), breast invasive carcinoma (BRCA), and thyroid carcinoma (THCA) showed prominent tumor–normal differences in UCP1 expression. In these cohorts, UCP1 expression generally decreased from normal tissues to early- and late-stage tumors, with the lowest levels observed in T3–T4 tumors. This trend suggests that loss of UCP1 transcript may accompany local tumor progression and increased invasiveness (Figure 4A,B). However, it must be explicitly noted that these are transcriptome-level associations and do not confirm the presence of functional UCP1 protein in tumor cells, nor is the causal direction established.

At the protein level, immunohistochemical studies have reported detectable UCP1 signal in subsets of colorectal, breast, lung, and ovarian cancers [111,112]. However, these observations are subject to several important confounders: (1) stromal or infiltrating adipocytes within tumor tissue can produce strong UCP1 signals that may be misattributed to tumor cells; (2) many commercial UCP1 antibodies exhibit cross-reactivity with other SLC25 family members, particularly UCP2 and UCP3, which are more widely expressed in cancer cells; (3) expression levels are typically orders of magnitude lower than in brown adipocytes, raising questions about functional sufficiency.

Mechanistic studies proposing tumor-cell-intrinsic UCP1 functions have largely relied on overexpression systems or pharmacological interventions with limited specificity. For example, the “tumor cell slimming” model proposed by Xiong et al. showed that activation of the PLCL1/UCP1 axis promotes a brown-fat-like lipid metabolic program in tumor cells and suppresses progression [113]. In triple-negative breast cancer, UCP1 overexpression was reported to inhibit malignant phenotypes by inducing mitophagy and pyroptosis [114]. While these findings demonstrate that ectopically expressed UCP1 can perturb cancer cell metabolism and survival when overexpressed, they do not establish that endogenous UCP1 executes these functions at physiological expression levels in primary human tumors.

#### 4.6.3. Tumor Immune Microenvironment: UCP1-Associated Immune Correlates

The relationship between UCP1 and the tumor immune microenvironment is also primarily mediated by systemic adipose–immune crosstalk. Thermogenic adipose tissue regulates systemic metabolism, adipokine secretion, inflammatory tone, and lipid availability, all of which can affect antitumor immunity. Tumor-associated adipocytes may provide fatty acids, cytokines, and extracellular matrix signals that support cancer progression, immune evasion, and therapy resistance.

To further explore the association between UCP1 and the tumor immune microenvironment, we estimated the infiltration abundance of 24 immune cell subtypes in the four representative cancer cohorts using the ssGSEA algorithm and analyzed their correlation with UCP1 expression (Table S3). The results revealed pronounced cancer-type heterogeneity rather than a uniform pan-cancer pattern:

In BRCA, UCP1 expression showed widespread positive correlations with most immune cell populations, with the strongest associations observed for innate immune subsets including mast cells, NK cells, and plasmacytoid dendritic cells, and a significant negative correlation with Th2 cells. This profile suggests that UCP1 may be associated with enhanced innate immune activation and attenuated Th2-skewed immunity in the BRCA microenvironment.

In THCA, correlations between UCP1 and most immune cell subsets were of low magnitude and generally lacked statistical significance, suggesting a limited immunomodulatory role of UCP1 in THCA.

In COAD, UCP1 expression showed the strongest positive correlation with macrophage infiltration, accompanied by a significant negative correlation with Th17 cells.

In STAD, the correlation profile was predominantly negative: UCP1 expression was inversely correlated with a broad range of T cell subsets, including Th17 cells, Tregs, Th1 cells, and CD8^+^T cells.

A consistent cross-cancer finding was the negative association between UCP1 and Th17 cells, which was replicated in THCA, COAD and STAD, pointing to a potential conserved role of UCP1 in constraining Th17-driven inflammatory responses. Overall, all correlation coefficients fell within the range of −0.27 to 0.27, representing weak-to-moderate transcriptome-level associations (Figure 4C). These findings only indicate correlation, cannot establish causality, and do not equate to the presence of functional UCP1 protein in immune cells.

A small number of studies have reported UCP1 expression in selected immune cell subsets under specific conditions. For example, a recent study reported that myeloid-derived suppressor cells in the tumor microenvironment reduce UCP1 expression to enhance their immunosuppressive activity [115]. However, these observations remain preliminary and have not been broadly replicated. In the thymus, earlier reports of immune cell UCP1 were later attributed to contaminating brown adipocytes surrounding the gland rather than to thymocytes themselves [30]. Whether UCP1 is functionally expressed in tumor-infiltrating immune cells at physiologically relevant levels therefore remains an open question requiring further validation with cell-type-specific purification and rigorous antibody controls.

Taken together, current evidence supports a dual and context-dependent role of UCP1 in cancer, with markedly different levels of validation. The systemic role of adipose tissue UCP1 in cancer cachexia and metabolic crosstalk is well established. In contrast, direct functions of endogenous UCP1 within tumor cells or tumor-infiltrating immune cells remain incompletely verified and should be interpreted with appropriate caution. Depending on tumor lineage, metabolic state, stromal composition, and disease stage, UCP1-mediated uncoupling may either restrain malignant progression when ectopically activated, or facilitate adaptation to oxidative and metabolic stress.

**Figure 5:**
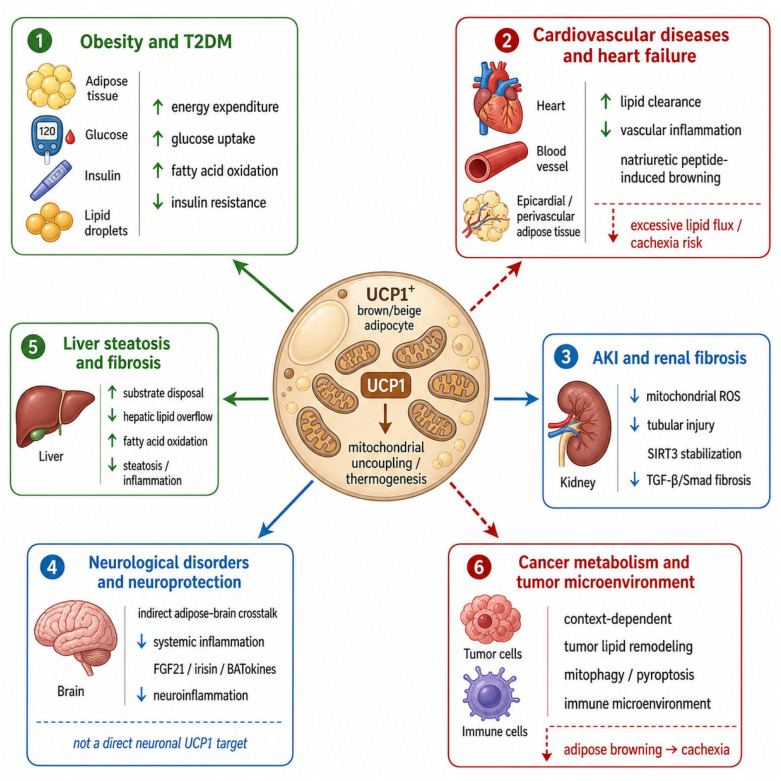
Proposed mechanisms linking UCP1-positive thermogenic adipose tissue to metabolic and organ diseases. UCP1-positive brown and beige adipocytes regulate systemic energy expenditure, substrate utilization, mitochondrial redox balance, endocrine signaling, and inflammatory tone. Through these mechanisms, BAT/beige fat may influence obesity and T2DM, cardiovascular disease, kidney injury and fibrosis, neurological disorders, liver steatosis and fibrosis, and cancer biology. Most effects are mediated indirectly through adipose–organ crosstalk rather than direct UCP1 expression in target organs. In cancer and advanced chronic diseases, excessive or maladaptive adipose browning may contribute to cachexia and energy wasting, indicating context-dependent effects of UCP1 activation. (Note: Green/blue solid upward arrows (↑): Increased physiological metabolic processes after UCP1 activation. Green/blue solid downward arrows (↓): Reduced pathological injury, lipid overload, inflammation or profibrotic signaling. Red dashed downward arrows: Harmful outcomes induced by excessive adipose browning).

Overall, UCP1 plays an important but highly context-dependent role across a broad spectrum of human diseases. UCP1-positive brown and beige adipose tissues contribute to the regulation of obesity, T2DM, cardiovascular disease, and hepatic steatosis, as well as to neurometabolic protection, by promoting energy expenditure, glucose and lipid clearance, mitochondrial homeostasis, and endocrine signaling. In kidney injury and fibrosis, UCP1-related pathways may exert protective effects by reducing mitochondrial ROS production and suppressing inflammatory and profibrotic signaling. However, UCP1 activation is not invariably beneficial. In heart failure, chronic kidney disease, and cancer cachexia, excessive adipose tissue browning and thermogenesis may exacerbate energy wasting, fat loss, and skeletal muscle atrophy. In cancer, UCP1 may inhibit tumor progression by enhancing lipid consumption, inducing mitochondrial stress, and promoting mitophagy or pyroptosis; conversely, under certain conditions, it may facilitate tumor-cell adaptation to metabolic stress. Therefore, UCP1 should not be viewed as a unidirectional therapeutic target, but rather as a key regulatory node linking adipose tissue metabolism, mitochondrial function, inflammatory responses, and inter-organ communication (Figure 5). To provide a structured overview of the evidence discussed above, we summarize the disease-specific evidence types, sites of action, human evidence levels, translational implications, and key limitations in Table 1.

## 5. Therapeutic Strategies and Translational Prospects for Targeting UCP1

Cold exposure and exercise represent the most physiological activators of BAT/UCP1 activity, but their clinical utility is constrained by poor compliance, interindividual variability, and limited long-term feasibility; moreover, the effect of exercise on human UCP1 induction is less consistent than in rodents. On the pharmacological front, dietary bioactive compounds constitute a broad class of candidate thermogenic modulators; however, their mechanistic links to UCP1 and translational evidence remain highly heterogeneous, as the vast majority act through upstream signaling nodes rather than direct UCP1 binding, and most data are derived from preclinical studies. Based on current evidence, natural product modulators of UCP1 can be stratified according to their mechanism of action and level of experimental validation.

### 5.1. Indirect UCP1 Regulators Targeting Upstream Thermogenic Signaling Pathways

Most natural products promote UCP1 expression and beige adipocyte formation by engaging conserved metabolic sensing and adrenergic signaling cascades. These compounds exert broad systemic metabolic effects, and their thermogenic activity cannot be disentangled from their other pleiotropic actions.

#### 5.1.1. β3-Adrenergic Signaling Mimetics

Capsaicin and capsinoids: As the most translationally advanced natural thermogenic agents, these TRPV1 agonists do not directly interact with UCP1. Instead, they activate gastrointestinal sensory nerves, triggering reflex sympathetic outflow to adipose depots and secondary activation of the β3-AR–cAMP–PKA cascade, which transcriptionally upregulates UCP1 and mitochondrial biogenesis [116]. In a randomized placebo-controlled human trial, daily oral intake of 9 mg capsinoids for 6 weeks increased supraclavicular BAT vascular density and showed a trend toward elevated resting energy expenditure, selectively in subjects with detectable baseline BAT activity [117]. However, the magnitude of effect is modest, and the contribution of UCP1-dependent versus other sympathetically mediated metabolic effects remains unquantified.

p-Synephrine: This proto-alkaloid from Citrus aurantium acts as a weak β3-adrenergic-like agonist. In mouse stromal vascular fraction-derived beige adipocytes, p-synephrine dose-dependently increased UCP1 mRNA and induced beige adipocyte morphology, effects that were abolished by β3-AR antagonist treatment [118]. All available evidence is limited to in vitro and rodent models; no human BAT activation data have been published.

#### 5.1.2. AMPK Pathway Activators

This is the largest and most mechanistically well-characterized class of natural thermogenic compounds, but AMPK is a ubiquitous cellular energy sensor, so all agents in this class are inherently pleiotropic.

Berberine: This isoquinoline alkaloid activates AMPK signaling in both white and brown adipose tissue, driving UCP1 upregulation, beige adipocyte recruitment, and increased whole-body oxygen consumption in diet-induced obese mice. A detailed mechanistic study further demonstrated that berberine promotes active DNA demethylation at the PRDM16 promoter via an AMPK-dependent increase in α-ketoglutarate, thereby reinforcing the brown adipocyte transcriptional program. In a small human trial in patients with non-alcoholic fatty liver disease, one month of berberine treatment (0.5 g three times daily) increased BAT mass and activity and improved insulin sensitivity. However, berberine also modulates gut microbiota, hepatic gluconeogenesis, and inflammatory pathways, so its metabolic benefits cannot be attributed specifically to adipose UCP1 activation [119].

Curcumin: In 3T3-L1 and primary white adipocytes, curcumin induces a brown-fat-like phenotype with robust upregulation of UCP1, PRDM16, and PGC1α, acting primarily through AMPK-dependent signaling. Dietary curcumin supplementation also reduced white adipose inflammation and increased BAT UCP1 protein in obese mice [120]. Curcumin has extremely low oral bioavailability and rapid systemic metabolism; there is no conclusive human evidence for BAT or UCP1 activation.

Quercetin and ginsenosides: Quercetin, ginsenoside Rg1, and ginsenoside Rb1 have each been shown to increase UCP1 expression and promote beige adipocyte formation in cultured adipocytes and mouse models via AMPK phosphorylation and downstream PGC1α/PRDM16 activation [121]. All data are preclinical, and none have been validated for thermogenic efficacy in humans.

#### 5.1.3. SIRT1 Activators and Mitochondrial Biogenesis Enhancers

Resveratrol: The canonical SIRT1 activator promotes mitochondrial biogenesis and upregulates UCP1 in rodent BAT via the SIRT1–PGC1α axis, increasing mitochondrial size, cristae density, and mtDNA content. Resveratrol also exerts broad anti-inflammatory, cardiometabolic, and direct antitumor metabolic effects independent of adipose tissue [122]. Its oral bioavailability is very low, and human studies have not consistently demonstrated BAT activation or UCP1-dependent thermogenesis.

Pentamethylquercetin (PMQ): This methylated quercetin analog exhibits improved bioavailability relative to the parent compound. In 3T3-L1 adipocytes and high-fat diet-fed mice, PMQ enhanced mitochondrial biogenesis, increased UCP1-positive multilocular adipocytes in white fat depots, and elevated whole-body thermogenic capacity via AMPK and PGC1α activation [122]. All evidence is preclinical.

### 5.2. Putative Direct UCP1 Ligands: Computational Predictions Without Experimental Validation

Recent advances in UCP1 structural biology have enabled in silico screening of natural compounds as potential direct UCP1 modulators. Using the cryo-EM structure of human UCP1 (PDB ID: 8J1N), molecular docking and molecular dynamics simulations have predicted that several natural products—including naringin, quercetin, curcumin, baicalein, rhein, and salsalate—can occupy the central nucleotide-binding cavity of UCP1 with favorable binding energies, potentially competing with inhibitory purine nucleotides to promote proton conductance [123].

However, these findings are strictly computational hypotheses and have not been validated by functional proton conductance assays, native mitochondrial studies, or structural determination of ligand-bound complexes. Whether these compounds achieve sufficient intramitochondrial concentrations in vivo to engage UCP1 remains unknown. Accordingly, in silico docking results do not constitute evidence of physiological UCP1 activation and should be interpreted as hypothesis-generating only.

### 5.3. Dual Metabolic Actions: Thermogenic Activation Plus Direct Tumor Metabolic Suppression

An emerging concept relevant to cancer metabolism is that many thermogenic natural products exert coordinated metabolic pressure on tumors through two parallel mechanisms: (1) systemic activation of adipose UCP1-dependent thermogenesis, which increases whole-body substrate consumption and reduces nutrient availability to tumors; and (2) direct inhibition of tumor-intrinsic metabolic pathways, including glycolysis, mitochondrial respiration, de novo lipogenesis, nucleotide biosynthesis, and glutamine anaplerosis.

For example, berberine, curcumin, and resveratrol each upregulate adipose UCP1 in preclinical models while simultaneously suppressing tumor cell glycolysis (via HK2, PKM2, or GLUT1 downregulation), inhibiting mitochondrial oxidative phosphorylation, and disrupting lipid and nucleotide synthesis pathways. This dual-action profile raises the possibility of synergistic anticancer efficacy, but it also further underscores the pleiotropic nature of these compounds: their biological effects cannot be ascribed to UCP1 modulation alone.

### 5.4. Small-Molecule Modulators and Structural Drug Design

A major goal is to develop small molecules that directly and selectively modulate UCP1 activity. Structure-guided drug design could exploit ligand-binding pockets, nucleotide-inhibitory sites, fatty acid interaction surfaces, or membrane lipid dependencies. However, direct UCP1 activation must be tightly controlled because excessive mitochondrial uncoupling may cause wasteful energy dissipation and impair cellular ATP homeostasis.

### 5.5. Translational Challenges and Limitations

Despite the growing interest in UCP1-targeted therapies, several fundamental challenges constrain their clinical translation. From a pharmacological perspective, the natural product thermogenesis field is limited by a lack of UCP1 specificity, as nearly all compounds act through broad upstream signaling nodes (AMPK, SIRT1, β-adrenergic signaling) rather than direct UCP1 binding, and their pleiotropic metabolic benefits cannot be attributed exclusively to UCP1-dependent thermogenesis. Moreover, the evidence base remains predominantly preclinical; only capsinoids and berberine have preliminary human data suggesting BAT activation, whereas most other compounds have been evaluated solely in cell culture and rodent models, often without adipose-specific loss-of-function controls to confirm UCP1 dependence. Pharmacokinetically, polyphenols, alkaloids, and other phytochemicals typically exhibit low oral bioavailability, rapid first-pass metabolism, and poor adipose tissue penetration, making it difficult to achieve the micromolar concentrations required for thermogenic effects in human adipose tissue at tolerable oral doses. Beyond these agent-specific limitations, several general barriers to BAT/UCP1-targeted therapy must also be considered. First, adrenergic regulation of thermogenic adipose tissue differs substantially between rodents and humans; while murine BAT is driven predominantly by β3-AR signaling, human BAT regulation is more complex, with β2-AR also playing an important role, and β3-AR agonists such as mirabegron often require relatively high doses that may produce cardiovascular sympathetic effects [124]. Second, the metabolic output of BAT is modest, contributing only ~5–10% of resting energy expenditure at baseline, with maximal acute stimulation adding less than 100 kcal/day, insufficient to drive clinically meaningful weight loss as monotherapy [125]. Third, human BAT activity exhibits marked inter-individual variability, influenced by age, sex, adiposity, ambient temperature, season, sympathetic tone, medication use, and metabolic disease status; it is more frequently detected in younger individuals, women, and those with lower adiposity, and declines with aging and obesity [4]. Collectively, these pharmacological, pharmacokinetic, physiological, and patient-level factors underscore the need for integrated strategies that combine selective UCP1 modulators, optimized delivery systems, patient stratification, and multimodal functional readouts in future clinical trial design.

## 6. Conclusions and Future Perspectives

UCP1 has evolved from a classical thermogenic effector of brown adipose tissue into a multifunctional regulator of mitochondrial bioenergetics, systemic metabolism, redox homeostasis, inflammation, and inter-organ communication. By dissipating the proton-motive force across the mitochondrial inner membrane, UCP1 drives adaptive thermogenesis and contributes to whole-body energy expenditure. Beyond this canonical function, accumulating evidence has implicates UCP1 in lipid and glucose metabolism, mitochondrial ROS control, immune remodeling, organ protection, and tumor biology.

Human genetic studies, BAT imaging, thermogenic gene signatures, and disease-associated expression profiles suggest that UCP1 may serve as a biomarker of metabolic health, disease susceptibility, and prognosis in selected pathological contexts. However, its clinical utility remains constrained by several limitations: ^18^F-FDG PET/CT only reflects BAT glucose uptake rather than direct UCP1 proton conductance and is confounded by glycemic status; adipose biopsy is invasive and unsuitable for routine dynamic monitoring; and no standardized circulating biomarker can reliably reflect endogenous UCP1 functional activity to date. Meanwhile, UCP1 expression in non-adipose tissues requires rigorous validation.

Despite recent advances, several critical questions remain unresolved. The precise molecular route of proton conductance, the dynamic interplay between fatty acids and nucleotides, the functional relevance of post-translational modifications, and the contribution of mitochondrial membrane lipids require further clarification. In addition, species differences, sex-specific responses, tissue heterogeneity, and UCP1-independent compensatory thermogenic pathways must be carefully considered when translating preclinical findings to humans.

Therapeutically, controlled activation of UCP1 or its upstream regulatory networks holds promise for obesity, insulin resistance, fatty liver disease, cardiovascular injury, renal fibrosis, neurodegeneration, and selected cancers. However, indiscriminate mitochondrial uncoupling may cause excessive energy expenditure, heat intolerance, or cachexia-like effects. Future strategies should therefore prioritize tissue-selective, reversible, and quantitatively controllable modulation of UCP1 activity.

With rigorous mechanistic validation and carefully designed clinical studies, UCP1 may become an important biomarker and therapeutic target in precision metabolic medicine.

## Figures and Tables

**Table 1:** Evidence-level summary of UCP1-related disease associations and translational implications.

Disease	Evidence Type	Site of Action	Human Evidence	Translational Implication	Key Limitation	Refs
Obesity & type 2 diabetes	Strong adipose-autonomous evidence; supportive human imaging/interventional evidence	Brown/beige adipose tissue (systemic metabolic regulation)	Moderate	May increase energy expenditure and improve insulin sensitivity and lipid handling	Limited BAT mass; indirect assessment of UCP1 activity; uncertain long-term efficacy	[8,62,63,64,65,66,67,68,69,70,71,72]
Cardiovascular disease & heart failure	Indirect adipose–cardiovascular crosstalk; preclinical and human observational evidence	Epicardial/perivascular & systemic BAT; no cardiomyocyte UCP1	Moderate (observational)	May improve lipid handling, vascular inflammatory tone, and cardiometabolic risk under controlled activation	Context-dependent effects; excessive sympathetic activation or adipose browning may aggravate wasting or lipid flux in advanced disease	[73,74,75,76,77,78,79,80]
Acute kidney injury & renal fibrosis	Preclinical evidence; indirect adipose–kidney crosstalk; speculative local renal evidence	Systemic BAT (indirect protection); renal UCP1 controversial	Low	May attenuate systemic oxidative and inflammatory stress in preclinical models	Endogenous renal UCP1 is unconfirmed; direct renal-cell function is unresolved	[29,33,81,82,83,84,85,86,87,88,89,90,91,92]
Neurological disorders & neuroprotection	Indirect adipose–brain crosstalk; preclinical and associative evidence	Systemic BAT (indirect crosstalk); no neuronal UCP1 in adult CNS	Low	May indirectly reduce metabolic and inflammatory risk factors relevant to neurodegeneration	No direct neuronal action; human causality remains unvalidated	[8,93,94,95,96,97,98,99,100,101]
Liver disease, steatosis & fibrosis	Indirect adipose–liver crosstalk; preclinical evidence; limited human metabolic evidence	Systemic BAT (substrate redistribution & adipokine effects)	Low to moderate	May reduce hepatic lipid overload and improve systemic metabolic status	Lack of hard liver endpoint data; quantitative contribution of BAT/UCP1 remains unclear	[59,102,103]
Cancer metabolism & tumor microenvironment	Adipose-tissue evidence in cachexia; exploratory pan-cancer transcriptomic correlation	Systemic BAT (nutrient competition); tumor cell UCP1 unvalidated	Low	May generate hypotheses for adipose–tumor metabolic crosstalk; inhibition of pathological browning may be relevant in cachexia	Tumor-intrinsic UCP1 is unproven; bulk transcriptomic signals may reflect adipocyte/stromal content or tumor purity	[30,104,105,106,107,108,109,110,111,112,113,114,115]

Notes: Evidence grading: Moderate indicates supportive human evidence, including imaging, observational, or interventional data, but not necessarily direct measurement of UCP1 proton conductance; Low indicates predominantly preclinical evidence, limited human validation, or mainly associative data. Evidence types were further classified as adipose-autonomous evidence, indirect adipose–organ crosstalk, preclinical evidence, associative human evidence, or speculative/incompletely validated local non-adipose UCP1 evidence. Abbreviations: UCP1, uncoupling protein 1; BAT, brown adipose tissue.

## Data Availability

No new data were created or analyzed in this study. Data sharing is not applicable to this article.

## Supplementary Materials

The following supporting information can be downloaded at https://www.mdpi.com/article/10.3390/ijms27146416/s1.
