# Supplementary material for: UCP1-Dependent Thermogenic Adipose Tissue in Human Disease: Adipose-Centered Mechanisms, Biomarker Limitations, and Translational Perspectives
Source: Int J Mol Sci. 2026 Jul 19;27(14):6416. doi: 10.3390/ijms27146416 (PMC13410023; doi:10.3390/ijms27146416)
Supplement: Supplementary file 1 [file ijms-27-06416-s001.zip › Supplementary Table Legends.pdf]

Supplementary Table S1. Pan-cancer expression summary of UCP1 across 33 TCGA cancer types.

Summary statistics of UCP1 expression [$\log_2$(TPM + 1)] in tumor and paired normal tissues across 33 cancer types from The Cancer Genome Atlas (TCGA). For each cancer type and group (tumor or normal), the table reports sample size (n), minimum, maximum, median, interquartile range (IQR), first quartile (Q1), third quartile (Q3), mean, standard deviation (SD), and standard error (SE). This table provides the underlying summary data for the pan-cancer expression radar plot shown in Figure 4A.

Supplementary Table S2. UCP1 expression stratified by pathological T stage in STAD, COAD, BRCA, and THCA.

Detailed descriptive statistics of UCP1 expression [$\log_2$(TPM + 1)] in normal tissues and tumor tissues stratified by pathological T stage (T1–T4) for stomach adenocarcinoma (STAD), colon adenocarcinoma (COAD), breast invasive carcinoma (BRCA), and thyroid carcinoma (THCA). For each subgroup, the table reports sample size (n), minimum, maximum, median, interquartile range (IQR), first quartile (Q1), third quartile (Q3), mean, standard deviation (SD), and standard error (SE). These data support the pathological T-stage association analyses presented in Figure 4B.

Supplementary Table S3. UCP1 expression, immune infiltration scores, and correlation matrices for STAD, COAD, BRCA, and THCA.

This comprehensive table contains three types of analytical outputs for each of the four cancer types (STAD, COAD, BRCA, THCA): (1) sample-level processed UCP1 expression values [$\log_2$(TPM + 1)]; (2) ssGSEA-derived relative infiltration scores for 24 immune cell subtypes (including aDC, B cells, $CD8^+$ T cells, cytotoxic cells, DC, eosinophils, iDC, macrophages, mast cells, neutrophils, NK cells, pDC, T cells, T helper cells, Tcm, Tem, TFH, Tgd, Th1, Th17, Th2, and Treg); and (3) Pearson and Spearman correlation coefficients between UCP1 expression and each immune cell type, along with corresponding test statistics and P-values. These data support the immune infiltration correlation analyses shown in Figure 4C.
